# Grossly Bloody Colostrum—The Need for Staff Education and Maternal Support

**DOI:** 10.3390/jcm12237326

**Published:** 2023-11-26

**Authors:** Katarzyna Wszołek, Stanisław Przewoźny, Adrianna Nowek, Angelika Odor, Paulina Małyszka (Hoffmann), Marcin Przybylski, Jakub Żurawski, Małgorzata Pięt, Maciej Wilczak

**Affiliations:** 1 Gynecology-Obstetrics Hospital, Poznan University of Medical Sciences, 60-535 Poznan, Poland; anowek@gpsk.ump.edu.pl (A.N.); mwil@ump.edu.pl (M.W.); 2 Department of Maternal and Child Health, Poznan University of Medical Sciences, 60-701 Poznan, Poland; 3 Students’ Scientific Association, Poznan University of Medical Sciences, 60-701 Poznan, Poland; stanislaw.przewozny.uni@gmail.com (S.P.); angelika.odor@gmail.com (A.O.); 4 Greater Poland Specialist Centre, 60-479 Poznan, Poland; przybylski@lutycka.pl; 5 Department of Immunobiology, Poznan University of Medical Sciences, 60-701 Poznan, Poland; zurawski@ump.edu.pl; 6 Facility of Practical Midwifery Teaching, Poznan University of Medical Sciences, 60-701 Poznan, Poland; mpiet@vp.pl

**Keywords:** lactation, breastfeeding, colostrum, human milk, grossly bloody colostrum, counseling, support

## Abstract

Background: Grossly bloody colostrum is defined as the presence of brownish or bloody-colored colostrum. The frequency is determined to be 0.1% of all births, and no predisposing factor for its occurrence was determined. The purpose of this study was to find factors that increase the probability of the syndrome and the possible influence of the presence of erythrocytes (red blood cells—RBCs) in breast milk on the symptoms of maternal milk intolerance in newborns. Methods: Here, 2 mL of colostrum was collected from 137 participants on postpartum days 1–3, separately from each breast, and transferred to the laboratory. For microscopic analysis, 0.5 mL of colostrum was centrifuged and then stained using the May–Grünwald–Giemsa method. Using an Olympus BX 43 light microscope at 400× total magnification, samples were visually assessed for the presence of RBCs. Additionally, the participants completed a questionnaire regarding their health status, observation of feeding tolerance of the newborn, observed milk color and other factors. Results: The number of RBCs in the yellow or white colostrum samples ranged from 8 to 1000 RBCs/mL and was found in 24.8% of cases. Regardless of the number of RBCs, the color of the milk was white (28.5%) or yellow (66.4%). Only one participant (0.7%) presented classical bloody-stained colostrum with visible bloody-milk colorization. We did not observe the influence of any analyzed factor as the reason for the increased number of RBCs in the colostrum. Conclusions: The presence of RBCs in breast milk did not cause milk intolerance among newborns and was independent of the analyzed factors. Mothers should be supported, and in view of the overall composition of breast milk, especially the content of immune cells in colostrum, even a temporary interruption in breast milk feeding in the case of grossly bloody colostrum is not recommended.

## 1. Introduction

Human milk, species-specific to the human mammal, has nutritional and non-nutritional functions and is characterized by its unique composition and distinctive properties, both for newborns born at term and prematurely [1,2,3,4,5,6,7]. The unique properties of this discharge are determined by a number of factors, including the presence of maternal blood cells and their varying concentration depending on the day of postpartum—the exceptional importance of colostrum is emphasized due to its concentrations in transitional and mature milk and the presence of immune cells [3,4,5,6,8,9,10], which are incomparable to those later. Stem cells and other cells of blood origin are also present in maternal milk [2,3,4,10,11,12]. The most extensively described cells in this group are those responsible for the immune system response, with the remaining cells described as “other cells with probable blood origin” [2]. RBCs may be qualified among them, and their high concentration in colostrum is considered a potential factor in changing the color of milk to brown or bloody, known as “rusty pipe syndrome” [13].

The mammary glands during pregnancy change under the influence of hormones. This is mostly due to estrogen acting to increase the flow of breast blood vessels, as well as the number of lobules, consequently, leading to their enlargement. The intense increase in breast volume significantly changes the shape of the myoepithelial cells surrounding the glands and milk ducts. Their walls become thinner, resulting in an increase in their permeability [14]. Under the influence of placental progesterone, the proliferation of milk glands occurs, and as a result of estrogen, a network of milk ducts develops. When the epithelium lining the milk glands transforms into a secretory epithelium, prepartum milk production begins—this usually occurs between 16 and 22 weeks of pregnancy. There is a small amount of it, which usually accumulates in the milk ducts, and, in some women, there is spontaneous leakage, which can be seen on the nipples [15].

Grossly bloody colostrum, described so far in the literature as “rusty pipe syndrome”, is generally described as a benign, painless, self-limiting occurrence of brown or bloody colostrum and a milk color change to yellow or whitish within 7 days of childbirth among most mothers [13,16,17]. This phenomenon was initially analyzed in a prospective study and described in 1990—the frequency was determined to be 0.1% of all births, and no predisposing factor for its occurrence was determined [13]. Microscopic examination of milk samples from mothers who had a change in the color of their milk to brown/bloody was found to contain RBCs and immune cells, and no atypical cells were observed [13]. It was pointed out that there was a gradual, spontaneous, usually bilateral change in the coloration of the milk from bloody to yellow/white in the following days after childbirth with no negative consequences for the mother or the newborn [13,14,15].

From the Polish perspective, the person who provides care to pregnant, giving birth and postpartum women are both midwives and gynecologists. Therefore, it is important to educate all caregivers about the possible phenomena that can occur during the normal lactation process.

The main aim of the study was to analyze the incidence of grossly bloody colostrum and the potential relationship between the total number of RBCs in colostrum and neonatal milk tolerance.

We would also like to propose a change in the nomenclature used for this phenomenon, from “rusty pipe syndrome” to grossly bloody-colored colostrum or blood-stained colostrum. The terminology used so far suggests the presence of tubes and rust in women’s breasts, which is not in line with current knowledge of the mammary gland and is negatively connotated—rust is associated with dirt, deterioration and something that should be cleaned and not used as long as it is rusty. Stopping the use of negative, dehumanizing terms (“rusts metal, machinery, equipment, not human beings”) seems to be crucial in building medicine and science based on respect for human beings, regardless of their gender.

## 2. Materials and Methods

This study was cross-sectional. Inclusion criteria were: early postpartum period, the colostrum presence (before the breasts engorgement) and sufficient supply of colostrum for the baby, especially in the case of mothers of premature babies. Four midwives collected colostrum between June 2021 and April 2022. Midwives spoke with mothers to explain the purpose of the study and the method of expressing colostrum and asked mothers to fill out the questionnaire. Mothers were aware that in the case of finding any atypical cells in their samples, they would be informed and the sample description would be provided.

After the written informed consent was signed by mother, approximately 2 mL of colostrum was manually expressed from 137 participants on postpartum days 1–3, separately from each breast, between the newborn’s feedings. Samples were numbered 1–137—every number was assigned to a particular participant. For the milk collection, we used sterile, single-use 3 mL syringes ENFitTM, intended for enteral feeding and closed it using a stopper from the same manufacturer. After the milk was expressed, every sample was preserved from temperature changes by putting it into polyurethane, insulated containers with a 4 °C temperature cold pack and immediately transferred to the laboratory. For microscopic analysis, 0.5 mL of colostrum was centrifuged (Cellspin II cytometer) for 10 min/500 rpm then stained using the May–Grünwald–Giemsa method. Slides prepared this way were labeled with the particular participant number. Using an Olympus BX 43 light microscope (Olympus, Tokyo, Japan) at 400× total magnification, samples were visually assessed for the presence of RBCs. The lower measurement limit was 0, but the higher measurement limit was 1000 because of the limitation of the method.

After explaining the purpose and conduct of the study to 140 participants, 3 women declined, 137 of them agreed to take part in the study, and written informed consent was obtained from those mothers. Participants were hospitalized in the postnatal wards in the Gynecology-Obstetrics Hospital, Poznan University of Medical Sciences, Poznan and postnatal ward in the Greater Poland Specialist Centre, Poznan.

Every microscope image and sample photograph published in this article came from the database collected during our study.

Additionally, the participants completed a questionnaire and assessed the following: their tendency to bruise easily, bleeding of the nose or gums (always, during pregnancy or none), nipple damage (laceration, scabs or no changes), Hoffman’s exercises during pregnancy, parity, whether they breastfed an older child if it was not their first pregnancy—if the mother observed the colostrum color if she breastfed another child (yellow, white, clear/transparent, green, brown, bloody or other)—observation of feeding tolerance of this newborn (the regurgitation range: seldom or more often—a few times per day or after every feeding), self-observed milk color after this childbirth (yellow, white, clear/transparent, green, brown, bloody or other), the color of the vomited stomach contents. The colostrum sample color was additionally assessed by the laboratory diagnostician (one person).

Statistical analysis was conducted using the statistical package R, version 4.0.5. Nominal variables were presented by the number of responses (percent of study group), while quantitative variables were presented as an arithmetic mean ± standard deviation (with normal distribution) or otherwise as a median (first quartile; third quartile). The normality of the distribution was checked using the Shapiro–Wilk test and by visual assessment of histograms. Due to the lack of a normal distribution, comparisons of RBC concentration against selected categorical variables were performed using non-parametric tests: the Mann–Whitney U test (comparison of 2 groups) or the Kruskal–Wallis test (comparison of more groups). Analysis of the relationship between RBC concentration and quantitative variables was performed using Spearman correlation analysis. A significance level of 0.05 was used in the calculations.

## 3. Results

Colostrum samples were collected from 137 participants on postpartum days 1–3. The mean maternal age was 31.38 years (±5.49), and 40.9% of mothers were primiparous. Among 24 mothers (17.5%), birth occurred prematurely (<37 weeks of pregnancy). Cesarean section was performed in 46% of analyzed cases, 49.6% gave vaginal, natural birth and in 4.4% of cases, a vacuum extractor was needed. Macroscopic change of colostrum color to red occurred in one case (0.7%). The number of RBCs in the remaining samples ranged from 8 to 1000 RBCs/mL and was found in 24.8% of cases. Regardless of the number of RBCs, the color of the milk was white (28.5%) or yellow (66.4%). Nipple cracking/damage was reported by 37.2% of mothers, 41.6% pointed to experiencing easy-bruising or easy-bleeding. Additionally, 35.8% observed occasionally occurring regurgitation among the newborns, Table 1.

We did not observe the influence of any analyzed factor as the reason for the increased number of RBCs in the milk. We did not find a statistical correlation between analyzed factors and total RBC samples, Table 2.

Due to the presence of a single sample of macroscopically blood-stained colostrum, it was not possible to perform statistical analysis in this case.

**Figure 1:**
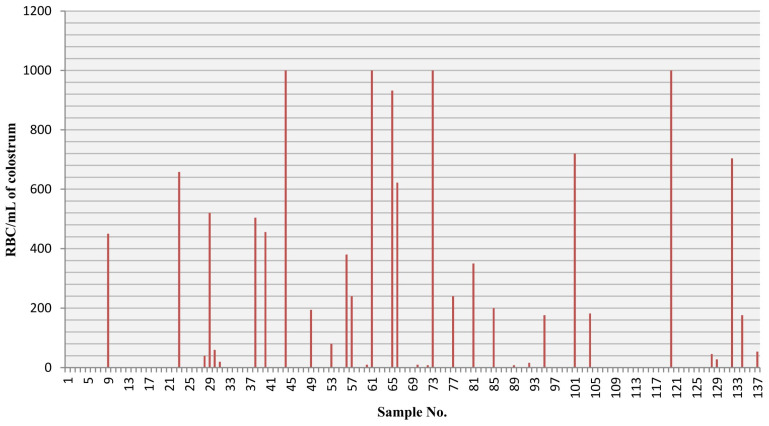
The total number of red blood cells (RBCs) in the colostrum samples in which any visible RBCs were found.

The distribution of RBCs (erythrocytes) per mL in the colostrum samples is presented in Figure 1.

**Figure 2:**
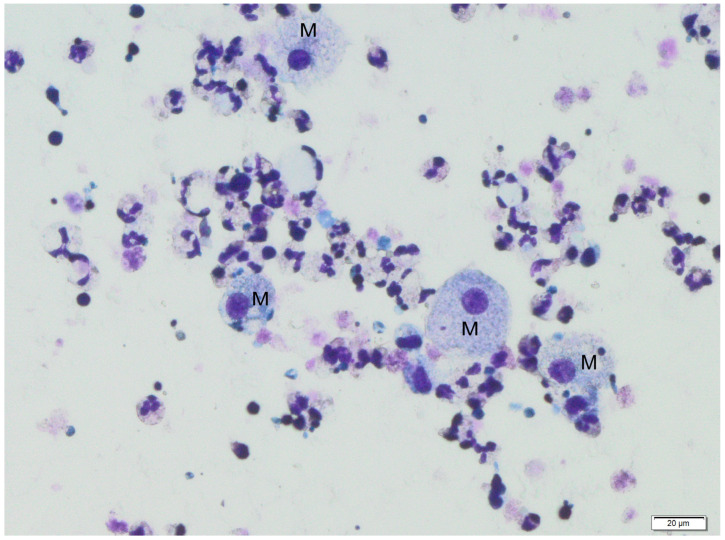
Human colostrum, magnification 400×. Macrophages (M) in the surroundings of other nucleated cells, mostly neutrophils. The figure represents the most common microscope image of colostrum samples of those we analyzed.

**Figure 3:**
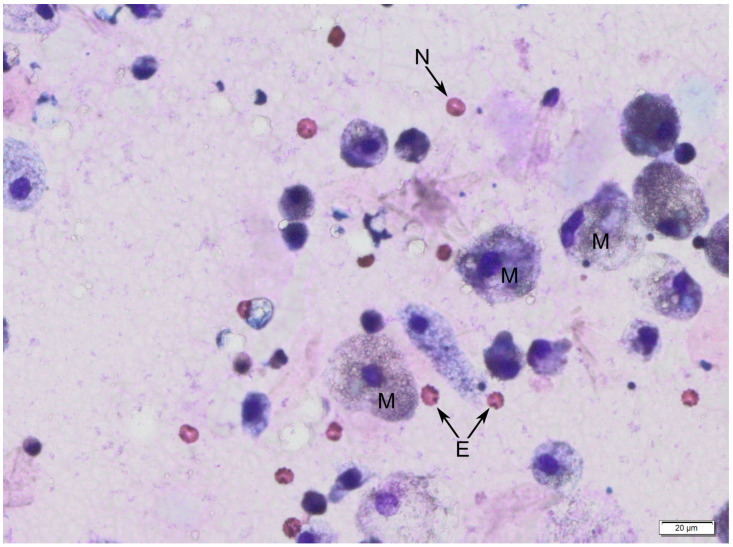
Human colostrum, magnification 400×. Macrophages (M) and red blood cells (RBCs) in two forms—echinocytes (E) and normocytes (N). The figure represents the most common microscope image of colostrum samples of those we analyzed.

The following figures show milk samples viewed under a microscope after they had been stained using the May–Grünwald–Giemsa method, Figure 2 and Figure 3.

**Figure 4:**
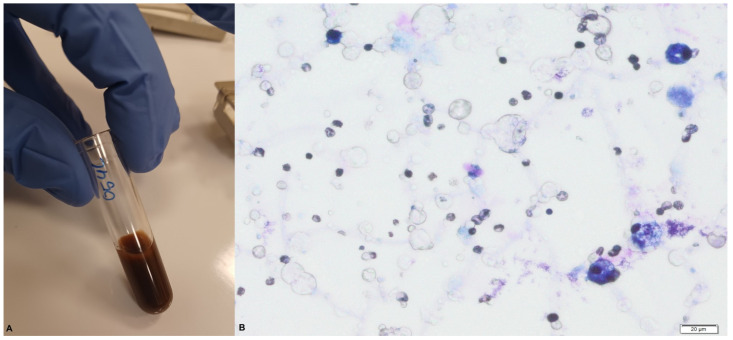
Blood-stained colostrum: (**A**) macroscopic and (**B**) microscopic views.

In the macroscopic blood-stained colostrum sample collected during our study (Figure 4), we observed > 1000 RBCs/mL.

During the analysis of slides, nucleated cells were recognized. The morphology of these cells suggests the presence of neutrophils, eosinophils, lymphocytes, macrophages and epithelial cells.

## 4. Discussion

The presence of RBCs in nipple discharge during pregnancy was described in 1990 by Lafreniere [16]. He presented other authors’ findings, which involved cytological assessment of nipple discharge and histological assessment of the tissue samples taken during biopsy in a group of women who had blood-colored discharge from their nipples [16]. In their findings, Kline and Lash [18] described the thin walls of capillaries presented in visible tissue spurs, penetrating up to the milk ducts. They assumed that in the case of these thin capillaries, wall rupture may yield a transudate of blood into the milk duct lumen [18]. In addition, in 1990, Merlob [13] reviewed the current state of knowledge in this field at that time and the management that was implemented when a bloody discharge from the nipples was found. He suggested, like other authors, that the possible cause was the milk expression, early breast engorgement or a tendency to mild bleeding among mothers who experienced this phenomenon in his research group.

According to Barco [14], the presence of blood in human milk may be the reason for gastrointestinal irritation among newborn babies. He suggested that when vomiting and regurgitation occur, formula feeding may be temporarily indicated. Newman and Pitman [19] believe that the blood present in the milk is not a threat to the mother or the baby but can irritate the baby’s stomach and cause regurgitation, but according to the authors, this is not an indication to stop breastfeeding [19]. In the group we analyzed, we also did not find any relationship between the number of RBCs and the mentioned complications or contraindications to feeding with maternal milk. Similar conclusions were stated by Tang et al. [15], who analyzed available literature data on the subject and 16 case reports of mothers who had bloody stained colostrum. Almost all of the women described whose milk was bloody were primiparous, including the participants in the group we studied. In the classic course of the syndrome, the bleeding is self-limiting from both breasts / multiple ducts, usually ceases by the seventh day after childbirth and is not accompanied by any other additional symptoms [15].

During our analysis, we found one classical grossly bloody colostrum case, but during the analysis, we found that among 24.8% of the analyzed colostrum samples, RBCs were present and, in some cases, the total amount was 1000 RBC/mL of colostrum. This is an interesting finding because those milk samples were yellow or white, and among those mothers, we did not observe brown or bloody milk colorization.

Smith et al. [20] found that human breast milk contains immune cells, and they presented neutrophils, small lymphocytes and macrophages. Further studies expanded the analysis of human breast milk and identified monocytes, T-cells, B-cells, NK-cells, neutrophils, eosinophils and immature forms, even stem cells [2,3,4,11,12]. Stem cells from human milk can differentiate in every cell among three germ layers. N. Goudarzi et al. [21] showed a significantly higher amount of these cells in colostrum in comparison to transitional and mature milk. Our study was not focused on the nucleated cells, but with the method we used, these cells were easily visible. The cells we identified are among the ones previously described. The most often noted cells were neutrophils, but we also found macrophages with sponge cytoplasm (foam cells) and lymphocytes. The morphology of some cells was changed too much to assess it properly. Li et al. [22], in their study of mother’s milk—colostrum (days 2–7), transitional (days 10–14), and matured—used a flow cytometer and noted the presence of leukocytes, monocytes, NK lymphocytes, B lymphocytes, T lymphocytes, cytotoxic T cells and helper T lymphocytes. The presence of all cells of the immune system in breast milk ensures the secretion of cytokines and the presentation of antigens for their further phagocytosis. The presence of these cells in milk makes it possible to effectively fight infections caused by various types of pathogens. The cells of the immune system present in the mother’s milk not only provide active immunity to the infant by producing bioactive ingredients but also modify the microenvironment of the newborn’s digestive tract [23,24]. A newborn’s immune system matures gradually. Critical early protection against many of the infectious diseases that the mother has previously experienced is provided by passive IgG antibodies delivered by the placenta and breast milk. On the other hand, milk contains all the cells of the immune system that are actively involved in the immune response [24].

The appearance of an intraductal papilloma in the breast may be the likely cause of blood in breast milk [25], and Wilson-Clay and Hoover state that spontaneous, profuse discharge from the nipples (clear or bloody) can be a warning sign of breast cancer [26]. For this reason, clinicians should be watchful, and if a participant reports the presence of a bloody discharge during pregnancy or after childbirth, an in-depth history should be taken, the mammary glands should be assessed by palpation, the phenomenon should be observed carefully and a diagnostic protocol implemented if the reason for bloody discharge is unclear [27]. We observed no atypical cells among the analyzed samples.

Other factors are known to change the color of human milk, such as the bacterium *Serratia marcenens* [28,29,30]. At room temperature, it produces a reddish-pink pigment (prodigiosin). This discoloration is often seen in bottles, on towels and breast pumps left overnight with milk residue. There are no clear recommendations for management. If there are no signs of infection in the baby, breastfeeding should be continued. To prevent the multiplication of bacteria, it is necessary to store the pumped milk properly and maintain hygiene in the use of the breast pump [28,29,30].

Case reports already published on “rusty pipe syndrome”, as well as our own experience, indicate a persistent fear or concerns among mothers and medical personnel regarding feeding the newborn with blood-stained colostrum [31,32,33,34,35,36]. A bloody colostrum composition analysis performed by Wszołek et al. [35] proved that the qualitative value of such milk was not changed. Taking into consideration colostrum values, even a short break in breastfeeding for no clear reason may be harmful for a newborn baby and is hazardous for the undisturbed lactation process [17,35,36,37,38]. This case was different than the one we reported previously [35].

It is within the competence of midwives in Poland to support mothers in the lactation process. Education during pregnancy and home visits after delivery are part of a community midwife’s work. During hospitalization, lactation support is provided by the staff of the hospital where the mother is staying. Depending on the financial resources of the hospital, assistance is provided by a midwife who takes care of the mother and child or by a midwife with additional courses and training employed as a lactation consultant. In hospitals with the third degree of reference, it is promoted to collect colostrum as soon as possible from the mother of a premature or sick baby after birth and to cover the mucous membranes of its oral cavity with maternal milk, regardless of the method of feeding [39].

## 5. Conclusions

Healthcare professionals should educate mothers during pregnancy about the different colors of colostrum. Mothers should know that the appearance of colostrum of a brown or blood color is not a contraindication to breastfeeding. Regardless of the number of erythrocytes in the colostrum, there was no statistically significant correlation between this fact and the incidence of regurgitation among newborns. In the case of grossly bloody colostrum, it is necessary to support the mother and observe the situation to see if the phenomenon resolves on its own. Prolonged bloody or brown coloring of breastmilk for more than 7 days or earlier alarming symptoms are an indication for diagnosis of breast disease, but the occurrence of this phenomenon in the first days after childbirth only occurs in a certain group of mothers and is not a pathological symptom. The most serious danger associated with a lack of education among medical staff and mothers is the temporary cessation of breastfeeding or inhibition of lactation and feeding the baby with formula. Given the unique composition of colostrum and breast milk, this intervention is unjustified and harmful.

## 6. Study limitations

Limitations include a small sample size, single setting and an absence of information on medicines taken by the participants and their diseases.

## Figures and Tables

**Table 1:** The characteristics of the study group.

Variable		Number of Women	Percentage
Maternal age, years (mean ± SD)		31.38 ± 5.49	
First pregnancy		47	34.3%
First birth		56	40.9%
Week of pregnancy (median; Q1; Q3)		Me = 39.00 (Q1 37.00; Q3 39.00)	
Birth mode	Cesarian section	63	46.0%
	Natural, vaginal birth	68	49.6%
	Vacuum extractor	6	4.4%
Gave birth prematurely (<37 week of pregnancy)		24	17.5%
A tendency of bleeding/bruising in the mother		57	41.6%
Nipple damage		51	37.2%
Regurgitation among the newborns		49	35.8%
Colostrum color	White	39	28.5%
	Red	1	0.7%
	Transparent	4	2.9%
	Green	2	1.5%
	Yellow	91	66.4%
Presence of RBCs in the colostrum sample	None	96	70.1%
	Present	34	24.8%
	Nondiagnostic specimen	7	5.1%
Grossly bloody colostrum presentation		1	0.7%

**Table 2:** Analyzed factors.

Variable	Number of RBCs in Analyzed Samples *	Statistics	*p*-Value
Colostrum color		U = 1342.00	0.107
White	0.00 (0.00; 104.00)		
Yellow	0.00 (0.00; 0.00)		
Nipple damage		U = 1756.50	0.112
No	0.00 (0.00; 0.00)		
Yes	0.00 (0.00; 43.00)		
Easy bleeding/bruising		U = 2159.50	0.513
No	0.00 (0.00; 12.50)		
Yes	0.00 (0.00; 0.00)		
Preterm birth		U = 1270.00	0.512
No	0.00 (0.00; 12.50)		
Yes	0.00 (0.00; 0.00)		
Newborn regurgitation		U = 1656.00	0.869
No	0.00 (0.00; 6.00)		
Yes	0.00 (0.00; 12.50)		
Parity (first pregnancy)		U = 1880.50	0.663
No	0.00 (0.00; 4.00)		
Yes	0.00 (0.00; 35.00)		
Mode of birth		χ2 = 1.98 df = 2	0.372
Cesarean section	0.00 (0.00; 0.00)		
Natural birth	0.00 (0.00; 22.00)		
Vacuum extractor	0.00 (0.00; 7.50)		

* Data for RBC levels are presented as median (first quartile; third quartile), comparison of groups using the Kruskal–Wallis test (birth method) or Mann–Whitney U test (otherwise). χ2—Kruskal–Wallis test statistics, df—degrees of freedom.

## Data Availability

The datasets used and/or analyzed in the study are available from the corresponding author upon reasonable request.

## List of Abbreviations

RBC	red blood cell (erythrocyte)
